# Participación social en el sistema de salud de Chile: aportes reflexivos desde la bioética

**DOI:** 10.18294/sc.2023.4486

**Published:** 2023-10-30

**Authors:** Camilo Guerrero-Nancuante, Andrea Melo, Paulina Gundelach, Nicolás Fuster

**Affiliations:** 1 Magíster en Salud Pública. Estudiante, Doctorado en Salud Pública, Escuela de Salud Pública Dr. Salvador Allende, Universidad de Chile. Académico, Escuela de Enfermería, Universidad de Valparaíso, Viña del Mar, Chile. camilo.guerrero@uv.cl Universidad de Chile Escuela de Salud Pública Dr. Salvador Allende Universidad de Chile Viña del Mar Chile camilo.guerrero@uv.cl; Escuela de Enfermería Universidad de Valparaíso Viña del Mar Chile; 2 Magíster en Enfermería. Académica, Escuela de Enfermería, Universidad de Valparaíso, Viña del Mar, Chile. andrea.melo@uv.cl Universidad de Valparaíso Escuela de Enfermería Universidad de Valparaíso Viña del Mar Chile andrea.melo@uv.cl; 3 Magíster en Enfermería. Académica, Escuela de Enfermería, Universidad de Valparaíso, Viña del Mar, Chile. paulina.gundelach@uv.cl Universidad de Valparaíso Escuela de Enfermería Universidad de Valparaíso Viña del Mar Chile paulina.gundelach@uv.cl; 4 Doctor en Ciencias Sociales. Académico, Escuela de Enfermería, Universidad de Valparaíso, Viña del Mar, Chile. nicolas.fuster@uv.cl Universidad de Valparaíso Escuela de Enfermería Universidad de Valparaíso Viña del Mar Chile nicolas.fuster@uv.cl

**Keywords:** Bioética, Participación Social, Sistemas de Salud, Chile, Bioethics, Social Participation, Public Health Systems, Chile

## Abstract

La participación social en salud se relaciona con la capacidad de intervención de los colectivos en el sistema sanitario. Desde la bioética, se ha enfatizado en la relevancia de la participación social en salud debido a los efectos positivos a nivel de los grupos sociales, de la estructura sanitaria y de los sistemas políticos democráticos. Para asegurar la participación social en salud, la bioética aboga por la incorporación de la deliberación como herramienta para la toma de decisiones vinculantes. El objetivo del presente ensayo es reflexionar sobre la participación social en la historia del sistema de salud de Chile desde la óptica de la bioética. Las principales reflexiones indican que la participación es de tipo consultiva, sin deliberación y, por tanto, sin distribución de poder. Asimismo, la participación social fue resignificada por la etiqueta de “ciudadana”, potenciando el carácter instrumental, individual y clientelar en salud. Para subvertir esta situación, se requiere incluir reflexiones bioéticas en la estructura sanitaria con el propósito que las comunidades puedan incidir de manera consistente en el sistema de salud.

## Abstract (en)

Social participation in health is related to the ability of collectives to intervene in the healthcare system. From a bioethical perspective, the relevance of social participation in health has been emphasized due to its positive effects at the level of social groups, the healthcare structure, and democratic political systems. To ensure social participation in health, bioethics advocates for the incorporation of deliberation as a tool for making binding decisions. The aim of this essay is to reflect on social participation in the history of Chile’s healthcare system from a bioethical perspective. The main reflections indicate that participation is consultative in nature, lacking deliberation and, therefore, the distribution of power. Additionally, social participation has been redefined under the label of “citizen,” promoting an instrumental, individual, and client-oriented character in healthcare. To subvert this situation, it is necessary to incorporate bioethical reflections into the healthcare structure to enable communities to consistently influence the healthcare system.

## INTRODUCCIÓN

La participación social ha sido objeto de análisis e interés de grupos académicos, sociales y políticos. Se define como la capacidad de los grupos humanos de organizarse para tomar decisiones frente a problemas u objetivos comunes^1^. En salud, la participación social se ha nutrido de las discusiones en torno a los derechos civiles y políticos de las personas. El concepto comenzó a tomar fuerza durante la Conferencia Internacional sobre Atención Primaria de Salud realizada en Alma-Ata, cuyo eje se relacionó en la interacción grupos sociales-sistemas de salud y el derecho de los colectivos organizados a codirigir las instituciones sanitarias^2^.

Desde la bioética, autores han reflexionado sobre la importancia de la participación social en relación con el diálogo colectivo, la incidencia (poder) que tienen los grupos sociales sobre la estructura sanitaria y el uso del espacio público para la legitimidad de los sistemas políticos democráticos. En esa dirección, se ha profundizado en la relevancia de la deliberación como herramienta democrática para la toma de decisiones colectivas, constituyéndose como un método racional y vinculante en los cursos de acción comunitarios. De acuerdo con lo anterior, la importancia de la deliberación radica en que los problemas colectivos pueden y deben ser solucionados por las personas afectadas. Por tanto, sin participación social deliberativa, los colectivos no logran solucionar sus problemáticas, lo que genera un aumento del malestar social^3^.

El sistema de salud de Chile ha presentado diversas transformaciones debido a los cambios políticos y sociales en la historia del país. Desde la Declaración de Alma-Ata, la participación social en el sistema de Salud de Chile ha transitado por diversas formas de involucramiento de las colectividades, siendo un fenómeno poco explorado desde la bioética. Por lo tanto, resulta relevante estudiar la participación social en el sistema de salud de Chile y los aportes reflexivos de la bioética dada la agudización de los conflictos sociales en los últimos años, la crisis de legitimidad de las instituciones públicas y la falta de reconocimiento de los colectivos como grupos activos en la resolución de los problemas sociales.

Con base en estos antecedentes, el siguiente ensayo tiene como propósito reflexionar sobre la participación social en la historia del sistema de salud de Chile desde la bioética. El texto se organiza en dos partes. La primera, desarrolla el concepto de participación social, luego la participación social en el campo sanitario y los aportes de la bioética en torno a la participación social en salud. La segunda parte analiza la participación social en la historia del sistema de salud de Chile, desde los aportes reflexivos de la bioética. El texto culmina con algunas propuestas de participación social en el sistema de salud que promueven el derecho de las comunidades a codirigir los espacios sanitarios, fortalecer los colectivos para la resolución de los problemas y, por tanto, promover la legitimidad de las instituciones públicas. 

## DESARROLLO

La participación social es un concepto amplio y sus interpretaciones responden a la heterogeneidad ontológica y epistemológica de quienes han logrado conceptualizarla. En el sentido más amplio, la participación social es entendida como el proceso en el cual un grupo de individuos logra organizarse e involucrarse en la toma de decisiones en un lugar determinado^4^. Por consiguiente, la participación social se relaciona con las acciones colectivas para la consecución de objetivos comunes y refuerza la idea de la inclusión de las personas como actores relevantes del entorno social.

La literatura señala distintas clasificaciones de participación social. Algunas perspectivas analizan la participación social desde el grado de involucramiento de los sujetos (activa o pasiva), según la territorialidad o membresía, de acuerdo con el nivel de participación en la toma de decisiones (vinculante y no vinculante) y según las formas de interlocución (institucional o no institucional)^5^.

A pesar de las diferencias analíticas, la participación social logró un estatus político-social a partir de los derechos civiles y los valores que la rodean, como es la responsabilidad colectiva, la justicia y la solidaridad^6^. Dada la universalidad de estos valores, intelectuales de gran parte del espectro político han incorporado el término participación social en sus despliegues discursivos^5^, pero con diversos matices según el rol que ocupan las personas en el proceso de participación. 

Aun cuando la participación social presenta una cierta noción de neutralidad, el concepto ha transmutado de acuerdo con las matrices políticas, económicas y sociales de cada territorio. A modo de ejemplo, los Estados liberales han realizado un giro al concepto de participación social, resignificándola a la etiqueta de participación “ciudadana”. La participación ciudadana se basa en que los individuos pueden construir sus destinos de acuerdo con sus capacidades individuales, siendo la participación una herramienta más en la convivencia civil^5^. Además, esta noción de participación incluye solo al segmento social considerado ciudadano, excluyendo a los grupos históricamente marginados. En contraposición, quienes abogan por concepciones más colectivistas entienden la participación social desde una perspectiva amplia, de pertenencia a un grupo social y de involucramiento activo en la obtención de objetivos comunes. Desde esta perspectiva, la participación social produce efectos importantes a nivel societal como es el respeto dialógico, la solidaridad y el fortalecimiento del tejido social^7^. 

### Participación social en salud

La participación social en el campo de la salud no está exenta de las disimilitudes presentadas anteriormente. En ese sentido, retomando los tipos de clasificación de participación social, se reconocen dos niveles: uno relacionado a la autogestión en salud (no institucional) y otra asociada a la participación con las estructuras sanitarias (institucional), es decir, en las interacciones entre el Estado y los grupos sociales^8^.

La literatura señala que la participación social en salud se sustenta en los derechos civiles y políticos de las personas, particularmente, en las libertades de los individuos de organizarse y participar del contexto social^8^. De esta manera, se produce un círculo virtuoso entre la acción colectiva de participación en el ámbito de salud y la interpelación al Estado en su rol garante de derechos sociales^8^.

El primer esfuerzo reflexivo de gran escala sobre participación social en salud se llevó a cabo en la Conferencia Internacional sobre Atención Primaria de Salud realizada en Alma-Ata en 1978. La síntesis de la Conferencia menciona que los “pueblos tienen derecho y deber en participar activamente en la planificación y atención en salud”^2^. De ello se desprende la apertura y capacidad que deben tener los sistemas sanitarios de garantizar la participación social, como también de relevar el rol de las organizaciones sociales en la toma de decisiones en salud. A partir de esto, la Declaración de Alma-Ata conmina a los Estados a promover la participación social mediante la descentralización y el control^2^, la descentralización respecto de dotar a los sistemas sanitarios de mayor dinamismo en sus respuestas clínicas y sociales, mientras que el control en dirección al empoderamiento de las comunidades locales^2^. 

Posteriormente, en la Conferencia Internacional sobre Promoción de la Salud en Ottawa, se reafirmó la necesidad de que las comunidades tuvieran participación efectiva en la toma de decisiones en salud^9^. Esta situación es concordante a lo descrito por Vázquez *et al*. que, citando a Foranoff, indica que la participación social en salud incluye los aspectos de planificación, implementación y utilización de los servicios de salud con el propósito de fortalecer la autonomía de los colectivos^10^.

Las últimas actualizaciones sobre participación social en salud se efectuaron por parte de la Organización Mundial de la Salud (OMS). Para 2005, la OMS estableció orientaciones para la participación social en salud con base en la comunicación de los colectivos, el trabajo en conjunto y el empoderamiento de la población^11^. A pesar de su relevancia, la participación social en salud ha quedado relegada a reformas sanitarias parciales, generando alejamiento de las fuerzas comunitarias en el sistema de salud, cuestión que se ha exacerbado debido a los efectos de las medidas de distanciamiento social durante la pandemia de covid-19^12^.

### Bioética y participación social en salud

Como se mencionó anteriormente, la participación social presenta múltiples reflexiones bioéticas que sustentan su aplicabilidad en el sistema de salud. La primera de ellas es la reflexión sobre la prioridad que tiene el diálogo colectivo en la participación social. En ese sentido, el filósofo chileno Humberto Giannini menciona que la acción comunicativa tiene profundas implicancias en la sociedad ya que cuando se genera diálogo, se reconoce moralmente al otro como sujeto^13^. Si se extiende esta reflexión filosófica a la participación social en salud, tanto las estructuras sanitarias (representadas por trabajadores/as de la salud) como los grupos sociales organizados, se reconocen moralmente unos a otros al momento de establecer puentes dialógicos en solución de problemas comunes o en la consecución de objetivos colectivos. 

La segunda reflexión se relaciona con la relevancia del poder en la participación social en salud. En esa dirección, Nora Donoso, citando a Michael Foucault, analiza la participación social como una redistribución del poder, cuyo accionar está determinado por la incidencia de los sujetos autónomos^14^. Por tanto, la participación social en salud contiene una dimensión de poder en cuanto a la incidencia de los sujetos, pues a mayor incidencia, mayor es la redistribución de poder. 

Un tercer elemento es lo mencionado por Hannah Arendt en cuanto al espacio público como lugar de participación social. Es en este espacio donde los individuos pueden interactuar y persuadir respecto a los problemas comunes, emitir juicios y demandar al Estado por mejoras en las condiciones de vida^15^. Por ello, la participación social se da en un espacio público, que evita potenciales coacciones políticas, económicas y sociales en el proceso de participación^5^. De acuerdo con esto último, la participación social en salud, siguiendo a Arendt, debería ocurrir en los propios territorios comunitarios y no en espacios ajenos a los grupos humanos organizados.

Por su parte, solo hace algunos años los problemas de mayor escala social han sido objeto de interés por parte de la bioética, al considerarse una disciplina relativamente nueva y focalizar su quehacer en los problemas individuales en salud. A juicio de Ferrer, las primeras etapas de desarrollo de la bioética se centraron en el bienestar individual, lo que produjo desinterés en los problemas sociales^16^. No obstante, la autora, citando a Wikler, refiere que actualmente la bioética se encuentra expandiendo su campo reflexivo a los problemas sociales en salud^16^.

Dado este interés emergente, algunos bioeticistas iberoamericanos han profundizado en el ámbito de la participación social y la bioética. En ese sentido, Diego Gracia refiere que la participación social debe ser un imperativo ético societal a propósito de la crisis de legitimidad en los distintos sistemas políticos democráticos^3^. De acuerdo con el autor, el principio básico de las democracias es la participación de los colectivos en el devenir social y en la co-construcción de objetivos comunes. Sin estos elementos, los sistemas sociales y las leyes tienden a beneficiar a unos pocos^3^. 

Lo interesante de las reflexiones de Diego Gracia es la inclusión del concepto de deliberación en el campo de la participación social como herramienta/instancia para el intercambio de opiniones, intereses y toma de decisiones. Por tanto, la participación deliberativa es de carácter vinculante, promueve la ética de los máximos (autonomía y beneficencia) y su marco de acción está delimitado por los mínimos morales (justicia y no maleficencia)^3^. 

En Chile, Miguel Kottow ha enfatizado en la necesidad de establecer reflexiones bioéticas a partir de los problemas sociales con el fin de evitar el instrumentalismo de la bioética pragmática^17^. En palabras de Kottow, los problemas sociales se exacerban cuando existe escasa interacción y participación social, generando sociedades con un alto enquistamiento de las personas. Esta situación resulta peligrosa cuando las sociedades son desiguales dado que las comunidades no encuentran espacios políticos para subvertir las condiciones inequitativas presentes en sus contextos. Para evitar esta situación, Kottow -citando a Schumpeter- plantea el cambio de las democracias elitistas por sistemas democráticos participativos y deliberativos^17^. 

Para superar las deficiencias de la bioética instrumental y de las democracias elitistas, Kottow propone la bioética pública. Este concepto se caracteriza por vincular la bioética con la realidad social y los valores colectivos a través de la deliberación^17^. Por tanto, la participación social y la deliberación son dos pilares fundamentales para la bioética pública. Respecto a la participación social, Kottow refiere su importancia a partir de la injerencia de las comunidades sobre “la cosa pública”, en la protección de los marginados, en el reconocimiento de la igualdad de los individuos que componen una comunidad y en la distribución del poder en las sociedades. Por su parte, la deliberación constituye un método racional para llegar a acuerdos a nivel comunitario y se desarrolla a través del diálogo sobre argumentos robustos y coherentes^17^. En síntesis, lo que añade la bioética al concepto de participación social es la deliberación. De acuerdo con Kottow, el fin último de la deliberación es fortalecer la participación de la sociedad para la elaboración de políticas públicas encaminadas al bien común.

### Participación social: trayectoria en el sistema de Salud de Chile

Esta segunda parte del ensayo profundiza sobre la participación social en la historia del sistema de salud de Chile y las reflexiones desde el campo de la bioética. Para comenzar, es relevante mencionar que los sistemas de salud no son ajenos a los cambios sociales y políticos de los territorios, es más, son estructuras que reproducen los problemas de la sociedad^3^. Siguiendo con lo anterior, la historia del país se ha forjado al alero de contradicciones de clase, acumulación de fuerza popular, golpes de Estado y -actualmente- en un proceso de profundización del neoliberalismo e intentos de resistencia, situación que ha generado cambios en la participación social en el sistema de salud.

Existieron varias instancias de participación social durante la historia del sistema de salud de Chile. La primera constatación de participación social, pero al margen del sistema de salud, fue la creación de las mutuales de seguridad. Estas instituciones solidarias, autogestionadas y con perspectiva de clase tuvieron como propósito agenciar y mejorar las condiciones de vida de las obreras y los obreros. La participación y autogestión en el ámbito sanitario fue una prioridad para la clase trabajadora y se vislumbró en la difusión de pasquines sanitarios como fue el caso de la “Hoja Sanitaria de la International World Workers” en la primera década del siglo XX^18^. 

Posteriormente, y ya con la institucionalización de los partidos políticos, la creación de los sindicatos, la promulgación del seguro obrero y la fundación del Sistema Nacional de Salud, la participación social se vinculó a la interacción entre las colectividades y el Estado. Para ello, el Estado tuvo que crear instituciones que permitieran canalizar la participación social en las estructuras de salud. En ese sentido, entre 1960 a 1973, se establecieron variadas estrategias para asegurar la participación social en salud a propósito de la alta acumulación de fuerzas populares en el escenario político. Una de ellas fue el programa de “Promoción popular” impulsado por el gobierno de Eduardo Frei Montalva durante su mandato entre 1964 y 1970. El programa tuvo como objetivo asegurar la participación social en las políticas estatales por parte de las organizaciones comunitarias y los sectores tradicionalmente marginados de los centros de decisión^19^.

En el marco del programa de “Promoción popular”, se crearon los consejos comunitarios de salud, a través del Decreto 250, de 1967^20^. Este Decreto estableció la creación de consejos comunitarios en cada establecimiento del Servicio Nacional de Salud y permitió la participación consultiva de las comunidades organizadas como Juntas de Vecinos, Centros de Madres, Clubes Deportivos, entre otros. Las acciones enmarcadas en los consejos comunitarios de salud fueron:

“…examinar los problemas de salud que afecten a la comunidad; propender a que ellos sean solucionados mediante acciones rápidas y eficaces; promover el interés de los habitantes para participar en forma activa en la solución de los mismos; colaborar en la divulgación de los planes y acciones de salud que programe la autoridad; representar las anomalías que aparezcan en la ejecución de esas acciones y, en general, procurar un mayor acercamiento de la comunidad con los establecimientos que ejecutan acciones de salud”.^20^

En paralelo a la creación de los consejos comunitarios de salud, en 1968 se promulgó la Ley 16880 sobre Organizaciones Comunitarias, cuya orientación fue generar las condiciones para que las comunidades pudieran interactuar con el Estado de manera permanente y dar respuesta a las necesidades sociales, incluidas las de salud^21^. 

Las políticas de participación social en salud se potenciaron bajo la presidencia de Salvador Allende, particularmente, con la creación de los consejos locales de salud a través del Decreto 602 del Ministerio de Salud Pública, en 1971^22^. En la práctica, los consejos locales de salud fueron las instituciones que reemplazaron a los consejos comunitarios con modificaciones en cuanto a la proporcionalidad paritaria de representantes de los trabajadores de la salud. Una característica relevante del Decreto 602 fue la eliminación del concepto “participación consultiva”, por lo que quedaba a la interpretación si los consejos locales de salud pudieran ser órganos vinculantes en las decisiones de los establecimientos sanitarios. Sin embargo, el Departamento Jurídico de la Contraloría General de la República hizo explícita la incompatibilidad del Decreto 602 respecto a la toma de decisiones vinculantes y se pronunció con relación a los Consejos Locales de Salud, definiendo que debían ser órganos asesores o consultivos^22^. En ese sentido, existieron al menos dos limitaciones en los consejos locales de salud. En palabras de Castañeda y Salamé, los consejos locales de salud estaban enmarcados en una política de emancipación social, pero fue contrastada por el caudillismo de dirigentes y el carácter de asesor al Servicio Nacional de Salud^23^. 

En consecuencia, desde la perspectiva bioética, la participación social en salud en los gobiernos de Frei Montalva y Allende presentó interesantes características al incluir un espectro social amplio para la gestión sanitaria y, por tanto, la expansión del rol político-social de los colectivos. No obstante, a pesar de la existencia de interacción entre los grupos sociales organizados y las estructuras sanitarias, la participación fue consultiva, situación que no permitió a las comunidades incidir de manera real en la toma de decisiones en el sistema de salud. Por consiguiente, no hubo deliberación social por parte de los involucrados en el ámbito de salud, cuestión fundamental cuando se vincula la participación social y la reflexión bioética.

En contraposición, la dictadura cívico-militar generó tortura, muerte y desaparición de miles de chilenas y chilenos y, con ello, la ruptura del tejido social. La intervención política por parte de la dictadura fue la instauración de la Constitución Política de la República de 1980, la cual señala el carácter subsidiario del Estado de Chile. Asimismo, esta carta magna aseguró la matriz económica neoliberal, reduciendo la capacidad estatal y dotando al sector privado de “libertades” para el desarrollo de negocios en el ámbito de derechos fundamentales como es el caso de la educación y salud^24^. Todo ello generó un cambio en la subjetividad de la población chilena, es decir, hubo transformaciones en la manera en que las personas representaron su entorno y cómo orientaron sus acciones prácticas. Es así como el neoliberalismo moldeó esta subjetividad en relación con las acciones prácticas que, en tiempos pasados, fueron consideradas constitutivas de la vida social de las personas: participación en barrios, generación de organización social, militancia en movimientos políticos, entre otros^25^. El sistema sanitario de Chile no quedó ajeno a este tipo de coacciones invisibles, produciendo sujetos clientes en salud^26^ y restringiendo severamente la participación social^27^.

Otro aspecto que afectó la participación social fue la visión sanitaria asistencial y la implementación de un sistema “mixto” de salud^28^. En cuanto a la visión sanitaria asistencial, se produjo la necesidad de atender los problemas de salud de manera individual y, junto al sujeto cliente en salud, llevó a la población chilena a preocuparse por cuestiones preferentemente clínicas. Las experiencias previas de involucramiento social con el sistema de salud fueron supeditadas por la posibilidad de acceder a la nueva institucionalidad privada en salud con la promesa de ser eficaz, pero sin atisbos de participación social en su estructura. Asimismo, la segregación social derivada del modelo neoliberal generó que las decisiones en el sistema de salud público sean tomadas por personas que, en su mayoría, utilizan el sistema sanitario privado, generando -como plantea Bauman en su tesis de “modernidad líquida”- un círculo no virtuoso de tomas de decisiones sesgadas^29^, por lo que la participación social no fue prioridad. 

Con lo señalado anteriormente, las consideraciones bioéticas sobre participación social en salud fueron anuladas por la brutalidad de la dictadura cívico-militar y las consecuencias del modelo neoliberal. Desde nuestra perspectiva, no existieron condiciones sociales y materiales para el ejercicio de la participación de las colectividades en el sistema de salud. En ese sentido, se han documentado ampliamente los afectos generados en este periodo histórico en la sociedad chilena a propósito del miedo a dar opiniones u organizarse con otras personas^30^. Sumado a nuevas subjetividades debido al sistema neoliberal, no se propendió a la participación social en salud. En consecuencia, durante este periodo no existió posibilidad de participación social, de diálogo, de reconocimiento de las comunidades, ni menos deliberación colectiva. 

Posterior al término de la dictadura cívico-militar, la Concertación de Partidos por la Democracia -coalición política que gobernó durante la década de 1990 y primera década del siglo XXI- no efectuó cambios en participación social en salud a excepción de la reforma al sistema sanitario en el 2005^31^ y la promulgación de la Ley 20500 sobre asociaciones y participación ciudadana en la gestión pública^32^.

La reforma de salud de 2005 contempló una serie de modificaciones al sistema sanitario, entre ellas, la participación social. De manera explícita, la reforma señala que la participación social se relaciona con “…dar respuesta a las expectativas de la población con participación y control social”^33^. Asimismo, abarcó los principios de universalidad, equidad, participación, derechos y deberes de los usuarios, entre otros^34^. 

Específicamente, en el punto de participación social -que posteriormente fue etiquetado como participación ciudadana- se conformaron tres dimensiones participativas: macro, meso y micro participación. En la actualidad y desde una perspectiva operativa, la participación social se efectúa en instancias nacionales, regionales y comunales, pero no son espacios vinculantes en la toma de decisiones, desfavoreciendo el empoderamiento y control social en salud^35^.

De igual manera, la Ley 20500 no cambió radicalmente los elementos de participación social expuestos en la reforma de salud. Tanto el marco normativo entregado por la Ley 20500 y como la Resolución Exenta 168 del Ministerio de Salud dictaminan que la participación es “ciudadana” y consultiva^32^, situación que desfavorece el involucramiento social en el sistema de salud. Por otra parte, Donoso detectó problemas adicionales en relación con la participación social en el actual escenario. A juicio de la autora, los problemas contingentes sobre participación social en salud se deben a la:

“…escasa renovación dirigencial, el ejercicio de liderazgos de carácter autocrático en algunas instancias, que ha ido generando una complicidad tácita entre los equipos de salud y los representantes de las comunidades locales, que contribuyen a la rigidización de los procesos de participación y la mantención del carácter instrumental y clientelar”.^14^

Con todo lo anterior, si bien se recuperaron espacios de participación social en salud perdidos en la dictadura, estos no presentaron cambios sustanciales para el empoderamiento colectivo y el control social efectivo. En ese sentido, el marco normativo y técnico actual carece de elementos bioéticos que promueven una sólida participación social en salud, por tanto, el esquema no incorpora cuestiones como la redistribución del poder, la deliberación colectiva y la incidencia de los colectivos en la toma de decisiones. Estas consecuencias fueron profundizadas por las fuerzas políticas hegemónicas respecto a la nueva denominación de participación “ciudadana”, potenciando la agencia individual de las personas en el sistema de salud y marginando a los grupos sociales considerados como “no ciudadanos”. 

## CONCLUSIONES

La participación social en salud es relevante para los Estados que buscan el bienestar colectivo. Desde la bioética se han planteado una serie de beneficios en distintos niveles, que incluyen el reconocimiento de los sujetos, la redistribución de poder y la legitimidad de lo público. En ese sentido, para una real participación social en salud se requiere de deliberación vinculante para la toma de decisiones, cuestión que resulta fundamental en el actual escenario caracterizado por la profundización del neoliberalismo, la atomización de las personas, la falta de credibilidad en la política y la disminución de la participación en el espacio público. 

La participación social en la historia del sistema de salud de Chile ha presentado una serie de limitaciones. Aunque se constata la intención de gobiernos proclives a la participación social, durante la historia del sistema de salud de Chile se ha perpetuado la participación de tipo consultiva, lo que ha generado una participación social desbalanceada en cuanto a la distribución de poder, paternalista y con escasa capacidad de decisión. La dictadura cívico-militar produjo severas restricciones en la participación social en salud a propósito de nuevas subjetividades (personas clientes en salud y miedo a organizarse colectivamente), desmantelamiento del Estado e irrupción de privados en el ámbito sanitario.

Por su parte, las fuerzas políticas actuales no han generado cambios sustantivos en el modelo social, político y económico. En el ámbito de la participación social han reconfigurado el concepto bajo la etiqueta “ciudadana”. Este nuevo consenso sobre participación presenta diferencias sustanciales con el concepto de “social”, tanto en el carácter constitutivo de la ciudadanía con base en la capacidad individual de agencia. Por tanto, el actual marco normativo-técnico de participación en salud no involucra cuestiones esenciales desde la perspectiva bioética, como es la deliberación en el ámbito sanitario y falta de articulación con las fuerzas vivas de los territorios.

Para subvertir las dificultades de la participación social en salud presente en nuestro contexto, resulta importante generar transformaciones en el sistema político-social-económico del país. Para cumplir con lo explicitado en la declaración de Alma-Ata, se deben generar las condiciones para un Estado sólido que responda a las necesidades en salud e incluya la participación social deliberativa como un eje importante en el desarrollo del sistema sanitario. En esa dirección, compartimos algunas sugerencias que podrían aportar en el campo de la participación social en el sistema de salud de Chile:

1. Cambios de las mallas curriculares en las profesiones de la salud, que incluya una profunda comprensión de la salud como un espacio de redistribución de poder y emancipación social. En ese sentido, resultaría interesante incorporar, de manera transversal en las asignaturas de pre y posgrado, conceptos y marcos que permitan profundizar de manera colectiva y transdisciplinar, aspectos fundantes de la participación social y bioética como es la deliberación colectiva en la gestión de los centros de salud.
2. Cambio del marco legal de participación (Ley 20500) que permita la deliberación y la participación vinculante. Esto aportaría mayor distribución de poder en los Consejos Locales de Salud para la cogestión sanitaria, en potenciales elecciones de autoridades unipersonales de centros asistenciales y en la creación de indicadores de salud que incluya la participación social como eje de evaluación de la estructura sanitaria. Además, resulta relevante que el marco de participación social en salud estipule mecanismos para evitar conflictos de intereses de los participantes, la cooptación de espacios participativos por los grupos políticos hegemónicos o el caudillismo de dirigentes.
3. Aumentar el presupuesto para la participación social en salud, que generaría condiciones materiales para llevar a cabo la vinculación de los colectivos en la estructura sanitaria. El presupuesto debería incluir gastos relacionados con la operacionalización de las instancias participativas e incorporar mecanismos para asegurar que las personas puedan participar activamente en sus territorios, es decir, liberar de tiempos tanto de trabajo remunerado como no remunerado para quienes asuman representación de sus comunidades en las estructuras de participación en salud.

Estas sugerencias recogen algunas reflexiones bioéticas respecto a la participación social en salud. Sabemos que la participación social se encuentra influenciada por múltiples dimensiones, sin embargo, la estructura sanitaria debería buscar los mecanismos para que el involucramiento de los colectivos en el ámbito de salud no sea meramente simbólico. Las transformaciones necesarias para asegurar una adecuada participación social en el sistema sanitario son de largo aliento, por lo que resulta un desafío continuar discutiendo y observando cómo las personas inciden de manera real en las estructuras sanitarias. A nuestro juicio, este desafío resulta decisivo para ampliar la discusión sobre los derechos colectivos y la legitimidad de lo público.

## References

[1] Sanabria Ramos G (2001). Participación social y comunitaria: Reflexiones. Revista Cubana de Salud Pública.

[2] Organización Panamericana de la Salud (1978). Conferencia Internacional de Atención Primaria de Salud, Alma-Ata, URSS, 6 al 12 septiembre de 1978.

[3] Gracia D (2001). Democracia y bioética. Acta Bioethica.

[4] Salas LM (1997). La participación social: una alternativa para la construcción social de la salud. Revista de Trabajo Social.

[5] Iturrieta Ruminado F (2008). Participación social y la nueva articulación entre Estado, mercado y sociedad civil.

[6] Centro de Investigación y Educación Popular (2015). Manual de ciudadanía y convivencia desde la construcción colectiva de sentidos y redes: Participación social y política.

[7] Menéndez E, Spinelli H (2006). Participación Social ¿Para qué?.

[8] Mercado G, Adarme V (2010). El enfoque de los derechos humanos en las políticas públicas: ideas para un debate en ciernes. Cuadernos CENDES.

[9] (1986). Carta de Ottawa para la promoción de la salud. Conferencia Internacional sobre la Promoción de la Salud: Hacia un nuevo concepto de la Salud Pública.

[10] Vázquez ML, Siqueira E, Kruze I, Da A, Leite IC (2002). Los procesos de reforma y la participación social en salud en América Latina. Gaceta Sanitaria.

[11] Egaña Rojas D, Iglesias Vejar L, Cerda Rioseco R, Molina Carrasco P, Gálvez Espinoza P (2020). Participación social en la atención primaria en salud: tensiones y contradicciones. Atención Primaria.

[12] Organización Mundial de la Salud (2022). Voz, agencia, empoderamiento: Manual sobre la participación social para la cobertura sanitaria universal.

[13] Ramírez Figueroa A (1997). Del bien que se espera y del bien que se debe de Humberto Giannini. Revista de Filosofia.

[14] Donoso N (2018). Tensiones y paradojas en los procesos de participación social en salud. Cuadernos Médico Sociales.

[15] Serrano C (1998). Participación social y ciudadana: Un debate del Chile contemporáneo.

[16] Ferrer M (2003). Equidad y justicia en salud: implicaciones para la bioética. Acta Bioethica.

[17] Kottow M (2011). Bioética pública: una propuesta. Revista Bioética.

[18] Fuster N, Moscoso P (2015). La hoja sanitaria, archivo del Policlínico Obrero de la IWW: Chile 1924-1927.

[19] Biblioteca Nacional de Chile, Memoria Chilena (2021). Ley de Promoción Popular.

[20] Chile, Ministerio de Salud Pública (1967). Decreto 250: Crea Consejos Comunitarios de la Salud.

[21] Chile, Ministerio del Interior (1968). Ley 16.880 de Organizaciones Comunitarias.

[22] Chile, Ministerio de Salud Pública (1971). Decreto 602: Crea Consejos Locales de Salud.

[23] Castañeda Meneses P, Salamé Coulon AM (2014). Trabajo social chileno y dictadura militar: Memoria profesional predictatorial: Participación social en salud, período 1960-1971. Rumbos TS.

[24] Chile (1980). Constitución Política de la República.

[25] Alemán J (2016). Horizontes neoliberales en la subjetividad.

[26] Gallo Acosta J, Quiñones Useche A (2016). Subjetividad, Salud mental y neoliberalismo en las políticas públicas de salud en Colombia. Athenea Digital.

[27] Méndez CA, Vanegas López J (2010). La participación social en salud: el desafío de Chile. Revista Panamericana de Salud Pública.

[28] Becerril-Montekio V (2011). Sistema de salud de Chile. Salud Pública de México.

[29] Hernández Moreno J (2016). La modernidad líquida. Política y Cultura.

[30] Waldman G (2014). A cuarenta años del golpe militar en Chile: Reflexiones en torno a conmemoraciones y memorias. Revista Mexicana de Ciencias Políticas y sociales.

[31] Urriola C, Infante A, Aguilera I, Ormeño H (2016). La reforma de salud chilena a diez años de su implementación. Salud Pública de México.

[32] Chile, Ministerio Secretaría General de Gobierno (2011). Ley 20500: Sobre asociaciones y participación ciudadana en la gestión pública.

[33] Chile, Ministerio de Salud (2012). Diseño e implementación de una metodología de evaluación, seguimiento y acompañamiento de la reforma de la salud de Chile.

[34] Infante A, Paraje G (2010). La Reforma de Salud en Chile.

[35] González J (2015). La participación ciudadana como instrumento del gobierno abierto. Espacios Públicos.

